# The design‐by‐treatment interaction model: a unifying framework for modelling loop inconsistency in network meta‐analysis

**DOI:** 10.1002/jrsm.1188

**Published:** 2015-11-20

**Authors:** Dan Jackson, Paul Boddington, Ian R. White

**Affiliations:** ^1^ Biostatistics Unit, MRCCambridgeUK

## Abstract

In this note, we clarify and prove the claim made Higgins *et al*. (2012) that the design‐by‐treatment interaction model contains all possible loop inconsistency models. This claim provides a strong argument for using the design‐by‐treatment interaction model to describe loop inconsistencies in network meta‐analysis. © 2015 The Authors. *Research Synthesis Methods* published by John Wiley & Sons, Ltd.

## 1 The design‐by‐treatment interaction model

Network meta‐analysis (Salanti, 2012) is a fairly recent development where data from more than two treatment arms are included in the same analysis and where all studies provide outcome data for at least two of these treatment arms. The consistency assumption underlies many network meta‐analyses. This assumption states that the relative treatment effect of *B* to treatment *A* plus the relative treatment effect of *C* to treatment *B* equals the relative treatment effect of *C* to treatment *A*. Because of the way in which study‐specific treatment effects are calculated, this assumption is necessarily true within studies that include treatments *A*, *B* and *C*. However, this assumption need not be true across the entire evidence network, even after accounting for between‐study heterogeneity.

We will use the term ‘design’, and the accompanying letter *d*, to refer to the set of treatments included in a study. For example, if the first design involves treatments *A* and *C* only, then *d* = 1 is taken to mean the ‘*AC* design’. This narrow definition of ‘design’ is specific to network meta‐analysis; the term ‘design’ has a much broader meaning in statistics more generally. Given this terminology, the design‐by‐treatment interaction model (Higgins *et al*., 2012) is

(1) $$\mu_{di}^{AJ}=\delta^{AJ}+\beta_{di}^{AJ}+\omega_{d}^{AJ}$$

for *J* = *A*, *B*, *C*, *D*, …, where $\mu_{di}^{AJ}$ is the true treatment effect of treatment *J* relative to the reference treatment *A* in the *i*th study of design *d*, *δ* ^*AJ*^ is the average (across all studies of all designs) effect of treatment *J* relative to treatment *A*, $\beta_{di}^{AJ}$ is a study‐by‐treatment interaction term to reflect between‐study heterogeneity and $\omega_{d}^{AJ}$ is a design‐by‐treatment interaction term to reflect inconsistency (variability between designs). We assume consistency within trials so that $\mu_{di}^{IJ}=\mu_{di}^{AJ}-\mu_{di}^{AI}$. The $\omega_{d}^{AJ}$, which we refer to as the inconsistency parameters, allow inconsistency within the network and mean that every design estimates a different set of treatment effects. If the inconsistency parameters in model (1) are treated as fixed effects, then they are not all identifiable. White *et al*. (2012) explain how to constrain some of the $\omega_{d}^{AJ}$ to zero in order to ensure that the model can be identified; Jackson *et al*. (2014) instead treat the inconsistency parameters as random effects, where the distributional assumptions made for the $\omega_{d}^{AJ}$ ensure that the model is identifiable.

### 1.1 Loop inconsistency models

The design‐by‐treatment interaction model was inspired by Lu and Ades' loop inconsistency models (Lu and Ades, 2006). Loop inconsistency models are intended to describe inconsistencies that may arise when closed loops resulting from studies of different designs appear in the network. Loop inconsistency models therefore appeal directly to our intuition about how inconsistencies in networks of evidence might arise. Having adopted a treatment ordering, *A*, *B*, *C*, *D*, …, Higgins *et al*. (2012) describe the assumptions made loop inconsistency models thus

‘⋅ all studies containing treatment *A* are assumed to estimate the same treatment effects;

⋅ all studies containing treatment *B* but not treatment *A* are assumed to estimate the same treatment effects;

⋅ all studies containing treatment *C* but not treatment *A* or treatment *B* are assumed to estimate the same treatment effects; and so on’

From this description, we can see that the types of design that multi‐arm studies (those that involve more than two treatment groups) are assumed to be consistent with depends on the treatment ordering. This means that the form of the loop inconsistency model depends on the treatment ordering. Higgins *et al*. (2012) claim that ‘The only model that contains all the Lu–Ades models (i.e. with all different treatment orderings) appears to be the design‐by‐treatment interaction model’. In this note, we prove this claim.

## 2 Lemma: Any given pair of designs is inconsistent in some loop inconsistency model

We begin by showing that any pair of designs is inconsistent in some loop inconsistency model. Establishing the impossibility of finding two different designs that are assumed to be consistent in all loop inconsistency models is a useful ‘stepping stone'to our Theorem below.

Let *S* be the set of *n* letters that denote the treatments included in the network meta‐analysis, and let *T* be the set of all subsets of *S* which contain more than one letter. For example, if *n* = 4, so that *S* = {*A*, *B*, *C*, *D*}, then the (2^*n*^ − *n* − 1) members of *T* are: *AB*, *AC*, *AD*, *BC*, *BD*, *CD*, *ABC*, *ABD*, *ACD*, *BCD* and *ABCD*. Hence the members of *T* are the possible designs in the network meta‐analysis. Next, there is also a collection of loop inconsistency models. A loop inconsistency model has an ordering of *S* and puts members of *T* into groups depending on the ordering of *S*. For example, the loop inconsistency model using the ordering *C*, *B*, *D*, *A* has three distinct groups of designs: Group 1 contains designs *AC*, *BC*, *CD*, *ABC*, *ACD*, *BCD*, *ABCD* because these are the members of *T* containing the first letter, *C*. Group 2 contains designs *AB*, *BD*, *ABD* because these are the members of *T* that contain the second letter, *B*, but not the first, *C*. Group 3 contains only design *AD* because this is the only member of *T* containing the 3rd letter, *D*, but not the first or second letters, *C* and *B*.

To prove the Lemma, we will call two members of *T* ‘separate’ if not all loop inconsistency models put them in the same group. Members of *T* that are separate are allowed to be inconsistent with each other in some loop inconsistency model. We claim that any two elements of *T* are separate. To prove this, let *P* and *Q* be different members of *T* (so that *P* and *Q* are different designs). Because *P* and *Q* are different, there must either exist a letter in *P* but not in *Q* or a letter in *Q* but not in *P*. Without loss of generality, suppose that the letter *Z* is in *P* but not in *Q*. Now, consider the loop inconsistency model whose order has the element *Z* first and then all remaining letters in alphabetical order. This loop inconsistency model sees *P* in the first group because *P* contains *Z*. However, this loop inconsistency model does not see *Q* in this first group (that contains *P*) because *Q* does not contain *Z*. Therefore, *P* and *Q* are separate.

### 2.1 Theorem: The ‘union’ of all loop inconsistency models is the design‐by‐treatment interaction model

There are *n* ! possible orderings of *n* letters, where each ordering can be used to define a loop inconsistency model. Higgins *et al*. (2012) observe that the ordering of the last two treatments is immaterial for the form of the loop inconsistency model, but this does not affect the result that follows. We put all the loop inconsistency models with *n* treatment groups into an arbitrary order, and we define *L*_*i*_ to be the set of groups resulting from the *i*th loop inconsistency model. For example, continuing with the example of the loop inconsistency model with ordering *C*, *B*, *D*, *A*, if this is the first loop inconsistency model, then we have

$$L_{1}=\left\{G_{1,1},G_{1,2},G_{1,3}\right\}\text{,}$$

where *G*_1,1_ = {*AC*, *BC*, *CD*, *ABC*, *ACD*, *BCD*, *ABCD*}, *G*_1,2_ = {*AB*, *BD*, *ABD*} and *G*_1,3_ = {*AD*}. Similarly, if the second loop inconsistency model has ordering *A*, *B*, *C*, *D*, then we have that

$$L_{2}=\left\{G_{2,1},G_{2,2},G_{2,3}\right\}\text{,}$$

where *G*_2,1_ = {*AB*, *AC*, *AD*, *ABC*, *ABD*, *ACD*, *ABCD*}, *G*_2,2_ = {*BC*, *BD*, *BCD*} and *G*_2,3_ = {*CD*}. The members of *L*_1_ and *L*_2_ are the sets of designs that are assumed to be consistent by the two loop inconsistency models and, for example, we can informally refer to the *i*th loop inconsistency model as the ‘*L*_*i*_ inconsistency model’. This is because the members of *L*_*i*_, which are *G*_*i*,1_, *G*_*i*,2_ and *G*_*i*,3_, describe which sets of designs are assumed to be consistent with each other in the *i*th inconsistency model. By assuming that all designs estimate different treatment effects, the design‐by‐treatment interaction model is now referred to as the ‘*T* inconsistency model’

We need to be explicit about what we mean by the expression ‘containing all loop inconsistency models’. To do this, we will begin by describing what we mean by an inconsistency model that contains multiple (but not necessarily all) loop inconsistency models: an inconsistency model is said to contain multiple loop inconsistency models if the sets of studies that it assumes to be consistent are also assumed to be consistent by all the loop inconsistency models that it contains. An inconsistency model is then said to contain all loop inconsistency models if the sets of studies that it assumes to be consistent are also assumed to be consistent by all loop inconsistency models.

We define the ‘union’ of two loop inconsistency models as the inconsistency model with consistency groupings described by *L*_*i*_ ∪ *L*_*j*_, where

$$L_{i}\cup L_{j}=\left\{G_{i,a}\cap G_{j,b},a,b=1,\cdots ,\left(n-1\right)\right\}$$

so that, for *n* = 4,

(2) Li∪Lj={Gi,1∩Gj,1,Gi,1∩Gj,2,Gi,1∩Gj,3,Gi,2∩Gj,1,Gi,2∩Gj,2,Gi,2∩Gj,3,Gi,3∩Gj,1,Gi,3∩Gj,2,Gi,3∩Gj,3}

and where any empty sets in *L*_*i*_ ∪ *L*_*j*_ are discarded. For our running example, this means that

$$L_{1}\cup L_{2}=\left\{\left\{AC,ABC,ACD,\text{ABCD}\right\},\left\{BC,BCD\right\},\left\{CD\right\}\left\{\text{AB},ABD\right\},\left\{BD\right\},\left\{\text{AD}\right\}\right\}$$

The ‘*L*_*i*_ ∪ *L*_*j*_ inconsistency model’ contains the ‘*L*_*i*_ inconsistency model’ and the ‘*L*_*j*_ inconsistency model’. For example, in the ‘*L*_1_ ∪ *L*_2_ inconsistency model’, if we further assume that {*AC*, *ABC*, *ACD*, *ABCD*}, {*BC*, *BCD*} and {*CD*} are consistent, and also that {*AB*, *ABD*} and {*BD*} are consistent, then we obtain the ‘*L*_1_ consistency model’. We conclude that we do not need the full design‐by‐treatment interaction model to contain the ‘*L*_1_ and ‘*L*_2_’ inconsistency models; rather, the ‘*L*_1_ ∪ *L*_2_ inconsistency model’ is sufficient for this purpose. Although the design‐by‐treatment interaction model contains the ‘*L*_1_ inconsistency model’ and the ‘*L*_2_ inconsistency model’, the ‘*L*_1_ ∪ *L*_2_ inconsistency model’ is a ‘smaller’ (or, in statistical parlance, reduced) inconsistency model that also contains these two inconsistency models. More generally, by the way in which the union of two inconsistency models is defined, the ‘*L*_*i*_ ∪ *L*_*j*_ inconsistency model’ contains the ‘*L*_*i*_ inconsistency model’ and the ‘*L*_*j*_ inconsistency model’ whilst assuming that as many different designs as possible are consistent with each other. We can therefore describe the ‘*L*_*i*_ ∪ *L*_*j*_ inconsistency model’ as the smallest inconsistency model that contains the ‘*L*_*i*_ inconsistency model’ and the ‘*L*_*j*_ inconsistency model’.

We define the union of more than two loop inconsistency models in the obvious way: *L*_*i*_ ∪ *L*_*j*_ ∪ *L*_*k*_ = (*L*_*i*_ ∪ *L*_*j*_) ∪ *L*_*k*_ = *L*_*i*_ ∪ (*L*_*j*_ ∪ *L*_*k*_). We have established in the Lemma that all pairs of designs are inconsistent in some loop inconsistency model. Hence, when we take the union of all loop inconsistency models, in order to obtain the smallest inconsistency model that contains all loop inconsistency models, we obtain the ‘*T* inconsistency model’ (the design‐by‐treatment interaction model). This is because if we instead obtained a reduced form of the design‐by‐treatment interaction model when taking the union of all loop inconsistency models, such as the ‘*L*_1_ ∪ *L*_2_ inconsistency model’, then we would require a pair of different designs to be consistent in all loop inconsistency models, and the Lemma establishes that this is impossible.

## 3 Conclusions

The union of all loop inconsistency models can also be conceptualised as starting with the consistency model (all $\omega_{d}^{AJ}=0$) and cumulatively introducing the inconsistency parameters for each loop inconsistency model in turn, where each newly introduced loop inconsistency model provides its inconsistency parameters as additional parameters to the model. We have established in this note that we require the design‐by‐treatment interaction model to contain all loop inconsistency models. Those who may find loop inconsistency models intuitively appealing could consider all possible treatment orderings and so fit all possible loop inconsistency models to their data. However, we can test the null hypothesis that there is no inconsistency in any loop inconsistency model by testing the null hypothesis that there is no inconsistency in the design‐by‐treatment interaction model. For example, using normal approximations and treating the inconsistency parameters as fixed effects, White *et al*. (2012) show how a global test for the presence of inconsistency can be performed. This work supports using the design‐by‐treatment interaction model is a unifying framework for modelling loop inconsistency in network meta‐analysis (Higgins *et al*., 2012).
